# Influence of lattice strain on Fe_3_O_4_@carbon catalyst for the destruction of organic dye in polluted water using a combined adsorption and Fenton process†

**DOI:** 10.1039/d0ra07866b

**Published:** 2020-10-26

**Authors:** D. Santhanaraj, N. Ricky Joseph, V. Ramkumar, A. Selvamani, I. P. Bincy, K. Rajakumar

**Affiliations:** Department of Chemistry, Loyola College Chennai 600 034 Tamilnadu India santhanaraj@loyolacollege.edu; Department of Polymer Science and Technology, Council of Scientific and Industrial Research (CSIR) – Central Research Laboratory Adyar Chennai 600020 Tamilnadu India; Catalytic Reforming Area, Light Stock Processing Division, CSIR – Indian Institute of Petroleum Dehradun-248005 Uttarakhand India; Department of Physics, MES College Nedumkandam Kerala 685553 India; Nanotechnology Research & Education Centre South Ural State University Chelyabinsk-454080 Russia

## Abstract

In this study, 8, 25 and 50 wt% Fe_3_O_4_@activated carbon (AC) catalysts were prepared by simple coprecipitation method. The efficiency of the catalysts for the advanced Fenton's oxidation process using methylene blue (MB) as a model substrate was tested. Both modified and unmodified activated carbon catalysts exhibited similar activity towards the Fenton's oxidation process. Therefore, it is difficult to identify the role of the catalyst in this dye removal process. Hence, we proposed a new methodology to remove the MB by adopting the adsorption process initially, followed by the Fenton's oxidation process. The proposed process significantly improved the methylene blue decomposition reaction over the 25 wt% Fe_3_O_4_@AC catalyst. However, this trend was not seen in pure activated carbon and Fe_3_O_4_@AC (8 and 50 wt%) catalysts due to the instability of the material in the oxidizing medium. The possible reason for the deactivation of the catalysts was evaluated from lattice strain calculations, as derived from the modified W–H models (Uniform Deformational Model (UDM), Uniform Stress Deformation Model (USDM) and Uniform Deformation Energy Density Model (UDEDM)). These results provided a quantitative relationship between the experimentally calculated lattice strain values and Fenton's catalytic activity. Furthermore, the optimized strain value and crystalite size of Fe_3_O_4_ on the activated carbon matrix are responsible for the high catalytic activity.

## Abstract 2

The activity of the catalyst was directly correlated using lattice strain calculations, as derived from the W–H model.

## Graphical abstract

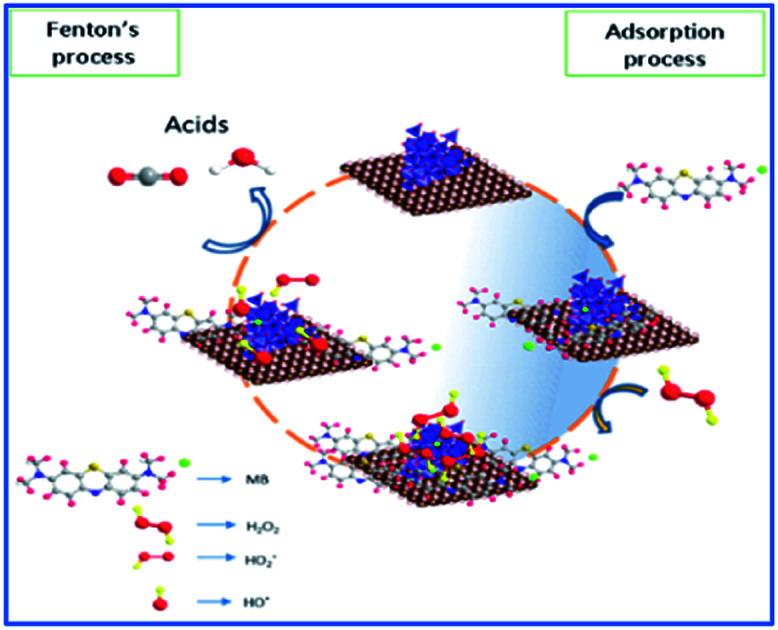

## Introduction

In recent years, uncontrolled natural and synthetic pollutants have increased greatly and lead to the decline of natural water sources. This created a huge demand for efficient technology to recycle the wastewater by a more economical route. Most commonly, the river bodies are being highly polluted by industrial and domestic organic chemicals such as textile dyes and other kitchen waste.^1^ As a result, many efforts have been taken to clean the organically polluted water by using simple and cost-effective technologies with less energy consumption. A detailed literature survey clearly shows that the advanced oxidation process can significantly improve the water quality either by photochemical or non-photochemical routes.^2–10^ Recent literature reports strongly suggest that photocatalysis is the best alternative method for removing organic pollutants by using various modified TiO_2_ materials and their related catalysts in the presence of harmful UV radiation sources. Hence, in order to avoid the UV induced photo reaction, the band gap energy of the photo catalyst has been tuned by several methods to work in the visible region.

Nevertheless, the visible light degradation methodology is not yet completely commercialized for cleaning the industrial wastewater due to the high permissible limit of organic contents and the lack of turn over frequency of the catalyst in the visible region.^11^ In general, advanced oxidation processes are eco-friendly and as well as more selective towards the complete oxidation reaction due to the formation of transitory radicals in the aqueous medium. In this context, the Fenton's oxidation reaction playing an indispensable role in the treatment of highly contaminated polluted water with powerful OH radicals, which have been generated directly from the decomposition of hydrogen peroxide over the Fe^2+^ ion sites at pH = 3.^12^ In recent years, the extensive research works have been focused for the development of heterogeneous photo-Fenton oxidation catalysts to operate at neutral pH medium. The major drawback of photo Fenton oxidation reaction is that it requires a transparent solution to UV or visible light radiation for an interfacial reaction; however, in most of the cases, the industrially polluted waters are not transparent to the light radiation and hence affects the degradation efficiency.^13^ Therefore, heterogeneous Fenton's oxidation reactions are still considered as a promising route for cleaning the organically polluted water even in the presence of dark environment. Notably, the iron-based catalysts such as Fe_3_O_4_, FeS_2_ and zero-valent-iron (ZVI),^14,15^ have been widely used for Fenton oxidation reaction. Among these series, iron oxide-based catalysts were extensively studied due to non-toxicity, simple preparation, and low cost. However, these materials are suffering to reach high efficiency due to the low surface area or bulk phase oxide formation. Recently, a variety of high surface area magnetic iron oxide materials were successfully employed for heterogeneous Fenton oxidation reaction including iron oxide supported on high surface CNTs and activated carbon materials.^16–18^ According to the literature, the common unavoidable problem in the Fenton oxidation reaction is that deactivation of catalyst and complicated regeneration technology was required. For instance, many studies are confirming that the catalyst deactivation was mainly occurred due to extended leaching of iron into the solution.^19^ In another study, Lejin Xu and Jianlong Wang concluded that the causes of deactivation was mainly due to surface oxidation of Fe^2+^ to Fe^3+^ by generated OH radicals during the course of reaction. Therefore, preserving the catalytic activity and stability is continue to be a challenging towards the Fenton oxidation reaction.^20^ Recently, hydroxylamine (HA) was used to prevent the oxidation of Fe^2+^ to Fe^3+^ in the surface bounded species, and they observed significant improvement for oxidative benzoic acid degradation in the presence of hydrogen peroxide.^21^ Despite these advantages, the use of HA may have a chance to pollute the water further, and hence the supplementary process is required to clean the wastewater. The simple adsorption method is an alternative route for the removal of dye molecules from the wastewater by using a variety of activated carbon material; however, this kind of adsorptive method is just transferring the pollutant from one phase to another, which is not desirable from the environmental point of view. Hence, in the present investigation, we proposed a combined strategy (adsorption and Fenton's oxidation reaction) using the inexpensive catalyst for continuous production of clean water from the dye polluted wastewater without involving any hazardous chemical regeneration treatment. To investigate the reaction pathway and possible reason for the catalytic deactivation, we have chosen MB as a model dye compound and studied in detail with the help of strain confinement effect.

## Results and discussion

### Determination of particle size and lattice strain

**Fig. 1:**
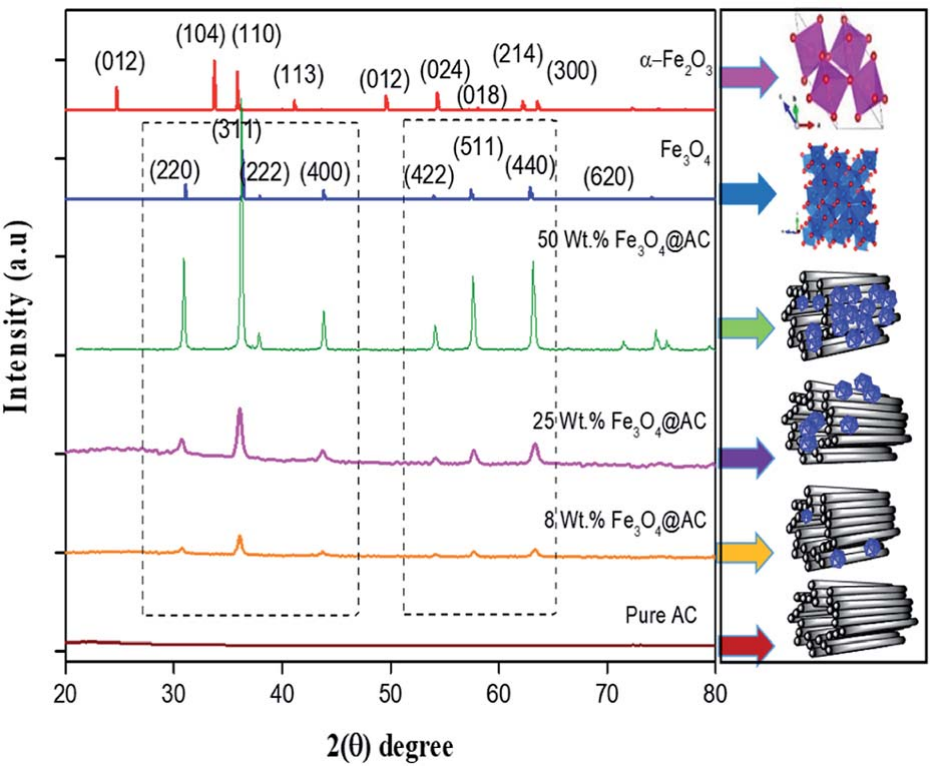
X-ray diffraction patterns of as-synthesized samples.

The crystallite size and phase formation of as-prepared (8, 25 and 50 wt% Fe_3_O_4_@AC) catalysts were verified by the powder XRD technique (Fig. 1). The peak positions at 2*θ* values of 30.7, 36.2 43.8, 54.1, 57.7, and 63.3 corresponds to the diffraction planes of (220), (311), (222), (400), (422), (511), and (440), respectively.^22^ These patterns were found to be in good agreement with previously reported diffraction values corresponding to the cubic spinel structure of Fe_3_O_4_ with a space group of *Fd*3*m* (227) according to the JCPDS card number 19-0629.^20^ Whereas, in the case of 8 wt% Fe_3_O_4_@AC, it displayed a weak diffraction pattern as compared to 25 wt% Fe_3_O_4_@AC catalyst indicating that the concentration of iron oxide was relatively lower than the 25 wt% Fe_3_O_4_ loaded catalyst. However, the phase formation on all the catalysts were still the same, demonstrating that the iron oxide loading does not affect the final crystal structure of the material. To further confirm any additional phase formation in the materials, the diffraction patterns are compared with the most stable phase crystalline structure (α-Fe_2_O_3_ JCPDS No. 13-534).^23^ The results from the XRD analysis showed that there was no evidence for the formation of α-Fe_2_O_3_ in the parent material representing that the materials exhibited good crystallinity structure. However, it is hard to ascertain from Fe_3_O_4_ material to γ-Fe_2_O_3_ by just comparing their diffraction patterns alone. Therefore, a special consideration is not required to distinguish both phases separately, since the gamma formation is unfavorable at low-temperature synthesis. Henceforth, we did not consider that particular gamma formation into the account for the Fenton's oxidation reaction. In addition to that, a small broad peak feature was also observed in the range 20–30° owing to the graphitic nature of activated carbon material as evidence from the unmodified XRD pattern (Fig. 1), which means that the structure of the activated carbon materials were not destroyed even after the modification. The average particle size of Fe_3_O_4_ loaded on the activated carbon matrix was calculated by plotting a graph between 1/*β vs*. cos(*θ*) from their respective FWHM of all the diffraction patterns, according to the Scherrer equation and the size of the particle is found to be 44, 99 and 232 nm, respectively. We observed that the size of the particle was nearly five times lower than that of pure Fe_3_O_4_ powder, which proves that the activated carbon matrix helps to decrease the particle size by their unique textural properties. Further, the accurate estimation of lattice constant derived from using Nelson–Riley (NR) extrapolation method in which both systematic and random errors can be minimized.^24^ The values of the lattice parameter obtained from each diffraction plane was plotted against the NR function (see Fig. S4†).1
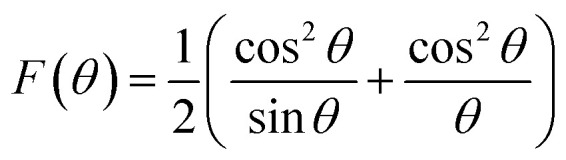
The extrapolation of the linear fit line to *F*(*θ*) = 0 or *θ* = 90° yields the accurate lattice constant, and the values are summarized in the Table 1. The unit cell parameter estimated for 25 and 50 wt% Fe_3_O_4_@AC, and Fe_3_O_4_ is found to be 8.449 Å, 8.343 Å and 8.659 Å, respectively. The reducing tendency of the unit cell parameter is observed mainly due to the decrease in the size of the particles, resulting in lattice contraction. Similar behaviour was also reported earlier.^25^ In general, the Scherrer formula will be adopted to measure the spherical shape size of the particle from the XRD peak broadening; however, it doesn't provide any information about the intrinsic lattice strain and stress within the crystal lattice. Usually, these kinds of stress and strain are mainly developed due to the existence of various types of defects present within the crystal lattice, as reported elsewhere.^26–28^

**Table 1:** Textural properties of the catalyst

Samples	Iron content^a^ wt%	Particle size (nm) (Scherrer method)	Unit cell parameter (Å)	BET surface area (m^2^ g^−1^)	Pore size distribution (nm)	Micro pore volume (cm^3^ g^−1^)	Meso pore volume (cm^3^ g^−1^)	Total pore volume (cm^3^ g^−1^)
Pure AC	—	—	—	960	2.02	0.201	0.616	0.900
8 wt% Fe_3_O_4_@AC	7.2	44	8.434	858	1.85	0.241	0.452	0.651
25 wt% Fe_3_O_4_@AC	24.2	99	8.449	669	1.53	0.22	0.301	0.513
50 wt% Fe_3_O_4_@AC	45.3	232	8.362	358	1.12	0.18	0.214	0.321
Pure Fe_3_O_4_	—	462	8.659	95	—	—	—	—

a Determined from ICP-OES analysis.

Many methods have been successfully developed to study the intrinsic strain in the crystal system but the W–H method is considered as the most appropriate and simple method for doing the calculations. In this connection, the strain as a function of support and non-support effect on Fe_3_O_4_ lattice deformation was calculated by W–H method, the obtained average particle size was compared with Scherrer's method. The observed results were summarized in Table 1. According to W–H method, the XRD line broadening was developed mainly due to the size effect of the particles or micro strain within the nano crystal. The XRD line broadening is given by the following eqn (2) as,

2 *β*_total_ = *β*_particle size_ + *β*_strain_

To fit into the uniform deformation model (UDM), modified W–H method, corresponding theta values are used as input values in eqn (3).3
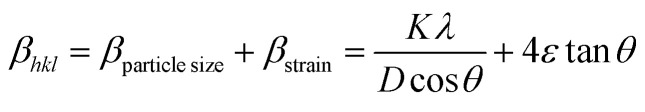


**Fig. 2:**
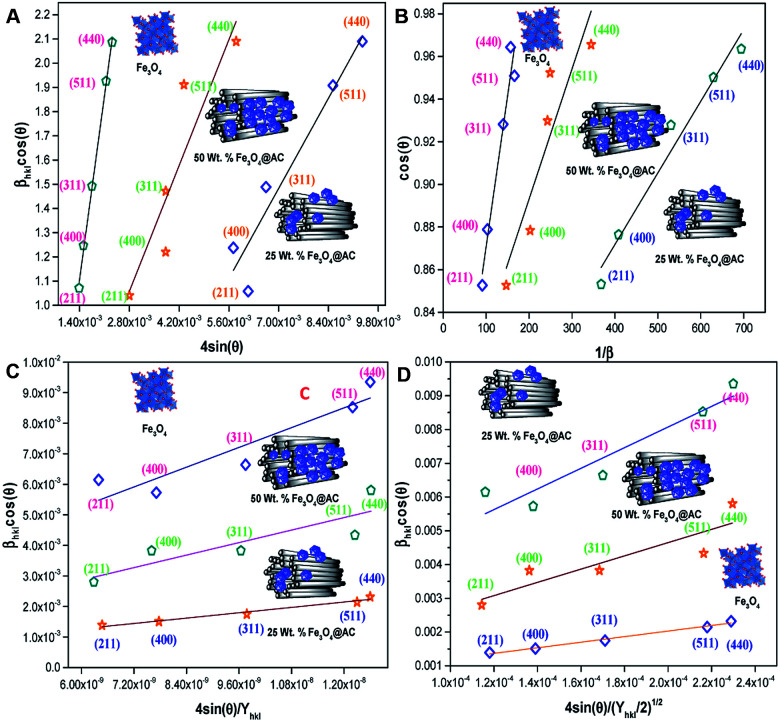
(A) Debye–Scherer equation plot, (B) *β*_*hkl*_ cos *θ vs.* 4sinθ (UDM) plots, (C) *β*_*hkl*_ cos *θ vs.* 4 sin *θ*/*Y*_*hkl*_ (USDM) plots and (D) *β*_*hkl*_ cos *θ vs.* 4 sin *θ* (2/*Y*_*hkl*_)^1/2^ (UDEDM) plots for pure Fe_3_O_4_, 25 and 50 wt% Fe_3_O_4_@AC.

The above equation was used to determine the particle size and as well as the uniform strain in the crystalline structure and it is commonly known as uniform deformation model (UDM). According to this model, the crystal system considered to be isotropic nature, where the direction of measurement is completely independent. Fig. 2 shows the plot of *β* cos(*θ*) against 4 sin(*θ*) corresponding to each diffraction patterns of iron oxide loaded on activated carbon and pure iron oxide catalysts. The slope and the intercept of the straight line provides the intrinsic strain and the average particle size of the catalysts, respectively. The plot showed a positive strain for both catalysts indicating that an increase in crystallite size might be due to lattice expansion. The calculated strain for 25 and 50 wt% Fe_3_O_4_@AC and Fe_3_O_4_ catalysts were found to be 3.50 × 10^−3^, 2.20 × 10^−3^ and 9.0 × 10^−4^, respectively. These values were compared as a function of support effect, interestingly the value of strain obtained from 25 wt% Fe_3_O_4_@AC catalyst was significantly larger than the pure Fe_3_O_4_ catalysts, it showed that as the particle size decreased, the lattice strain was dramatically increased. In the previous report, iron oxide nano particles were prepared by using with and without capping agents, and the particle size and lattice strain was calculated using W–H method. As summarized in Table 2, the comparison results are showing that although the particle size of 25 wt% Fe_3_O_4_@AC catalyst was relatively higher than the reported value, the strain *ε* value has unusually increased. It indicates that the development of strain owing to the confinement of iron oxide particle inside the porous architecture. The particle size and strain values predicted from the UDM model based on the assumption we made that the crystal has homogeneous and isotropic nature. Nevertheless, it does not actually fit into the real crystal. In general, most of the crystals are anisotropic, and hence, the modified W–H model (USDM) is required for calculating the stress and strain in the crystal lattice.^29^

**Table 2:** Geometrical parameters of the catalysts

Samples	Scherrer method	Williamson–Hall method								
		UDM		USDM			UDEDM			
	Size *D* (nm)	Size *D* (nm)	Strain *ε* (10^−3^)	Size *D* (nm)	Strain *ε* (10^−3^)	Stress *σ* (MPa)	Size *D* (nm)	Strain *ε* (10^−3^)	Stress *σ* (MPa)	Energy density (kJ m^−3^)
25 wt% Fe_3_O_4_@AC	99	72.3	3.50	69.3	3.35	537	72.9	3.43	549	941.5
50 wt% Fe_3_O_4_@AC	231	198	2.20	174	2.13	341	198.2	2.19	351	384.6
Fe_3_O_4_	462	346	0.90	345	0.908	145	346.6	0.92	147	67.8
FcP NPs	9.10	11.10	2.88	N/D	N/D	N/D	11.10	2.88	350	503.7 (ref. 26)
FC-NPs	10.00	11.25	1.53	N/D	N/D	N/D	11.25	1.53	273	208.5 (ref. 26)

According to the Hooke's law the stress and strain can be expressed as follows

4 *σ* = *ε* × *Y*_*hkl*_

5
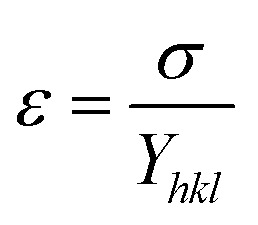


According to USDM, the broadening of XRD patterns are mainly transpired due to the development of stress in the crystal structure and the anisotropic nature of Young's modulus.^30^ On substituting the value of *ε* in eqn (3) and the model equation is given by below:6
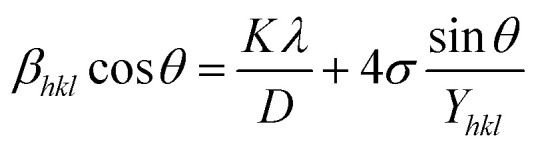


For a cubic crystal, Young's modulus is expressed by the following relation as,^31^7
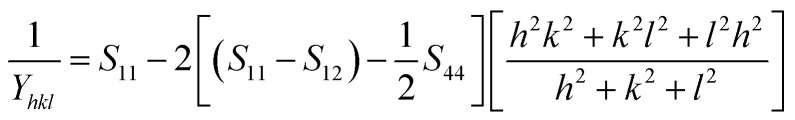
where *S*_11_, *S*_12_, *S*_44_ are known as elastic compliances of cubic Fe_3_O_4_ particles, and it can be derived from the elastic stiffness constants *C*_11_, *C*_12_ and *C*_44_, respectively, which are as follows:8
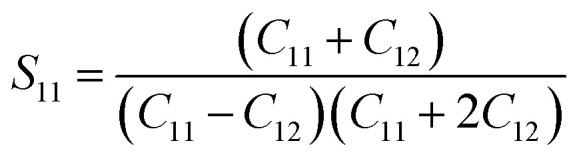
9
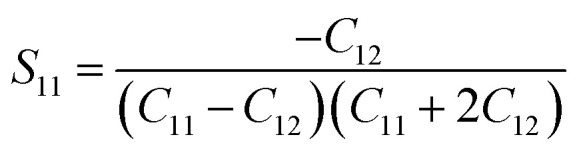
10
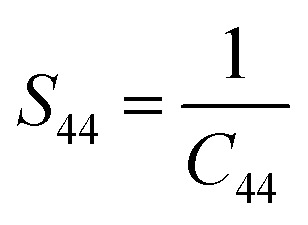


The value of elastic constants (Gpa) at ambient pressure for *C*_11_, *C*_12_, *C*_44_ (cubic Fe_3_O_4_) is 260.6, 148.3 and 63.3, respectively.^32^ By substituting these stiffness constant values in eqn (8)–(10), the elastic compliances values have been calculated as 0.00655, −0.00238 and 0.01580, respectively. From the above values, Young's modulus has been calculated from (220), (311), (400), (511) and (440) diffraction peaks as 165.6, 160.5, 152.6, 156.0 and 165.6 Gpa, respectively. Fig. 2 shows a plot of *β*_*hkl*_ cos *θ* against 4 sin *θ*/*Y*_*hkl*_. The value of stress and particle size calculated from the slope and the intercept of the equation. The results are summarized in Table 2. According to the Hooke's law, the USDM model is assumed as a linear relationship between stress and strain; however, in many real crystal systems, a linear proportionality between stress/strain and isotropic nature alone cannot be considered for understanding the structural deformation. Since, it exhibits different types of defects, dislocation, and agglomerates; it may create the imperfection in most of the crystal systems. To decipher the uniform anisotropic lattice strain in all directions of the crystal and their respective lattice strain in the crystal, the UDEDM model will be employed. According to Hooke's law, the energy density (*u*) is directly proportional to strain, and it can be expressed as follows:11
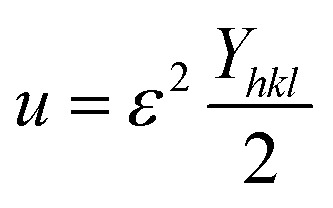
where *Y*_*hkl*_ is the anisotropic Young's modulus. Then eqn (3) can be rewritten according to strain and energy,12
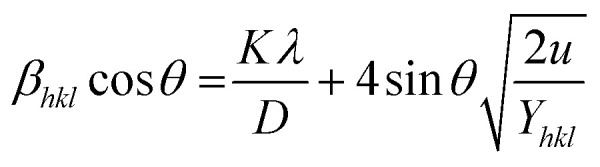


**Fig. 3:**
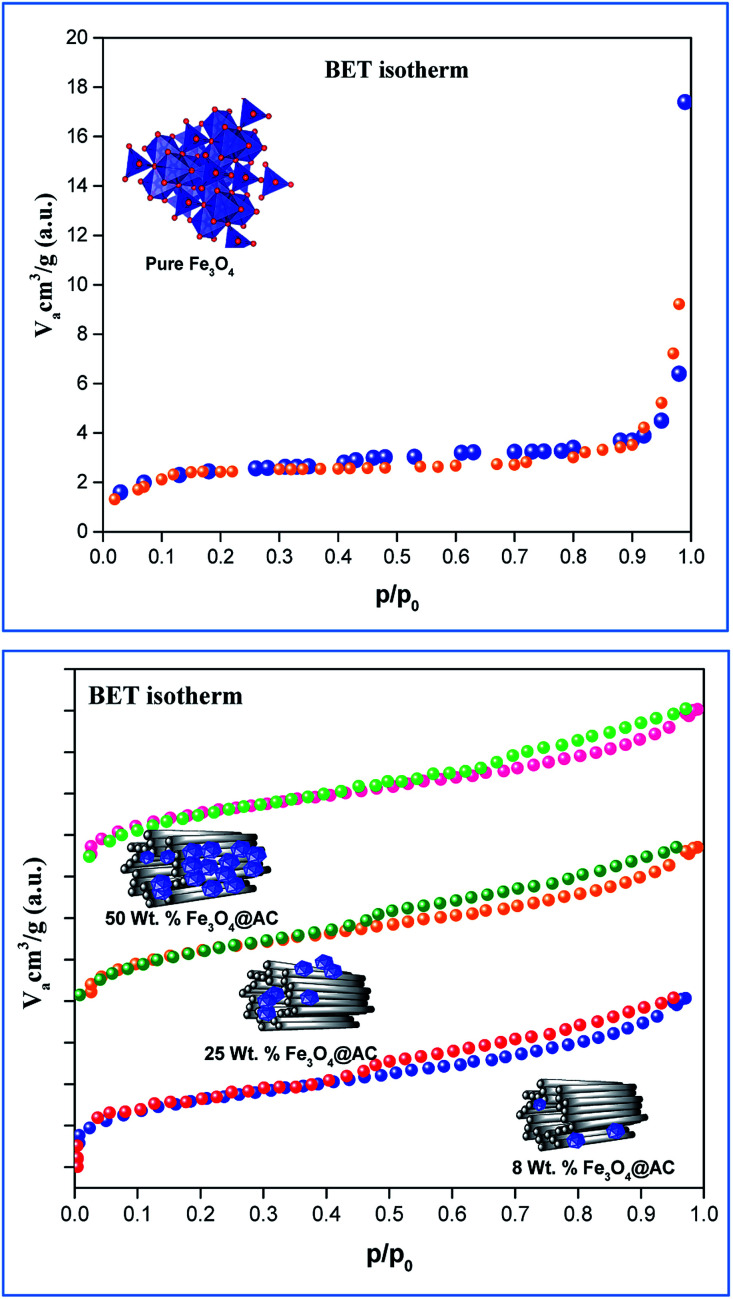
Nitrogen adsorption/desorption isotherms of catalysts.

The straight-line equation is popularly known as uniform deformation energy density mode (Fig. 2). From the linear fit model, the uniform deformation energy density of the crystal can be calculated by plotting the value of *β*_*hkl*_ cos *θ* in *x*-axis and 4 sin *θ*/(*Y*_*hkl*_/2)^1/2^ in the *y*-axis. The intercept of the line provides the average size of the particles, whereas the slope of the equation gives the energy density value in terms of kilojoules per unit volume. As compared to pure iron oxide particle, the calculated density energy was significantly higher for iron-oxide loaded activated carbon catalyst than pure iron oxide catalyst indicating that iron was embed in the more strain environment. These results are in good agreement with our lattice parameter calculation (Table 1). In fact, the values were relatively larger than the reported value confirming that the materials possess more defective sites, which can be achieved by simple precipitation method. The overall detail particle size and crystalline study data concluded that most of the iron oxide was mainly located inside the pores of activated carbon material, and it might be responsible for the higher uniform deformation energy density in the overall crystal system. Moreover, it is noted that there is a significant difference between the value of particle size predicted by the Debye–Scherrer method and from the three W–H models such as UDM, USDM, and UDEDM, respectively. In the Debye–Scherrer model, the particle size was determined from the XRD line broadening, which mainly contributed from the dependencies angle, whereas in the case of the modified W–H method, the particle size determination was accountable by both strain and size induced width to the total broadening. Further, the textural properties of the pure Fe_3_O_4_ and Fe_3_O_4_ supported activated carbon catalysts were characterized by using N_2_ adsorption–desorption measurements (Fig. 3), and their values are summarized in Table 1. The pristine Fe_3_O_4_ exhibits type I isotherm, while iron oxide loaded catalysts displayed a combination of type I and IV according to IUPAC classifications.^33^ The obtained results were compared with commercially available pure activated carbon.^34^ As summarized in Table 1, the textural properties such as surface area, pore diameter, and pore volume was dramatically decreased after the loading of Fe_3_O_4_ in the activated carbon matrix; however, the microporous region has not been affected significantly. The decreasing tendency clearly explains that most of the iron oxide is located in the mesoporous region rather than in the microporous architecture structure.

**Fig. 4:**
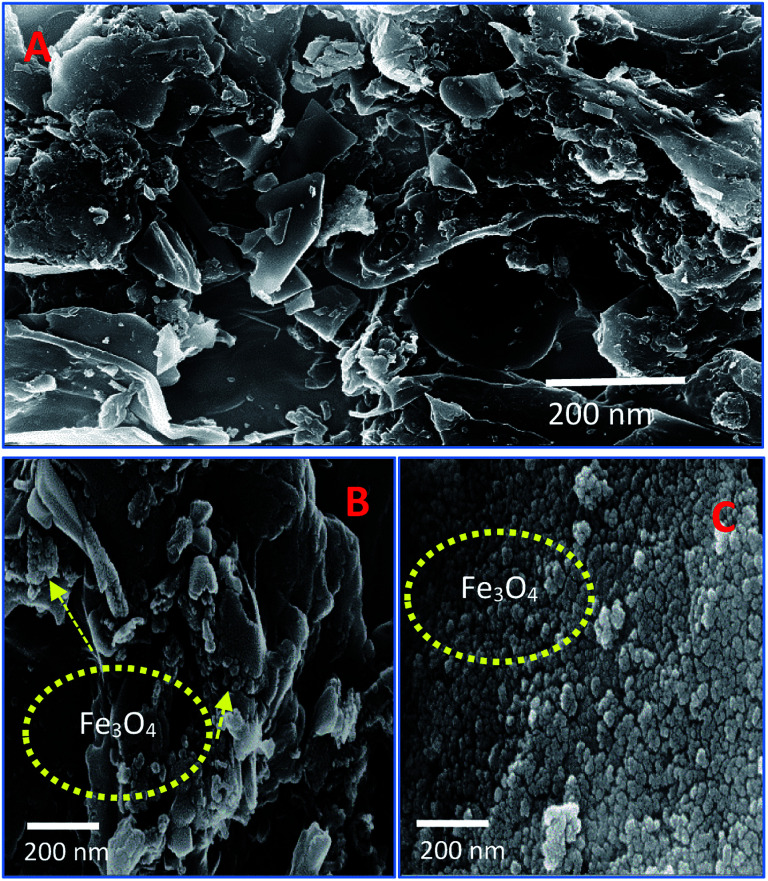
SEM images of (A) pure Fe_3_O_4_, (B) 25 wt% @Fe_3_O_4_ and (C) 50 wt%@Fe_3_O_4_ catalysts.

**Fig. 5:**
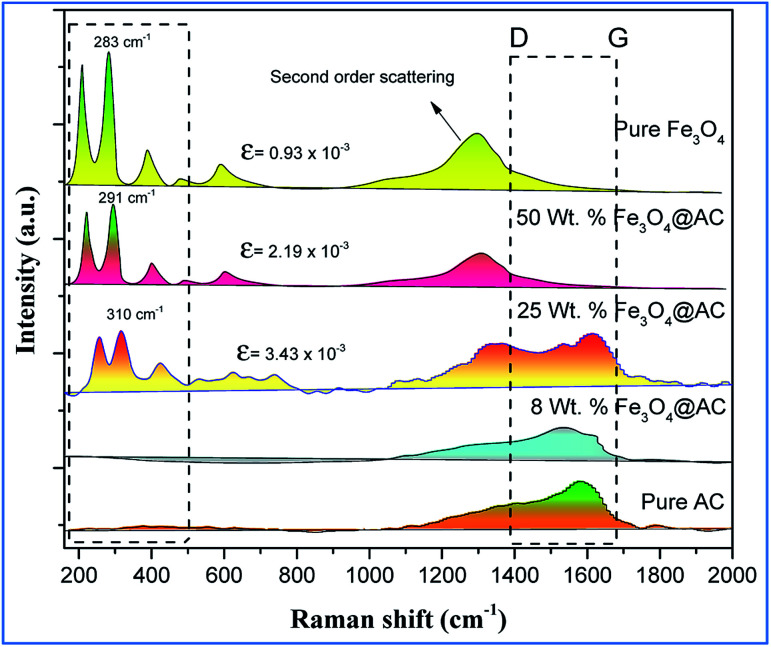
Raman spectra of pure and modified Fe_3_O_4_ catalysts.

The scanning electron microscope images are clearly showing that pure activated carbon surface has irregular morphology. The agglomerated spherical shape of Fe_3_O_4_ particles are clearly visible on 25 wt% @AC catalyst, and the location was highlighted in the image (Fig. 4b). This situation was dramatically improved on 50 wt% @AC catalyst of the catalyst where the activated carbon surface was completely covered with iron oxide particles. As shown in Fig. 5, the pure and modified Fe_3_O_4_ on carbon materials exhibit specific Raman bands at the range from 250 to 800 cm^−1^, which are the characteristic feature of Fe_3_O_4_ particles.^35,36^ Interestingly, a significant shift in the Raman signals was observed for all iron oxide modified catalysts as compared to pure Fe_3_O_4_, in particular, its influence was more on 25% Wt. Fe_3_O_4_ catalyst. The shift in peak position is caused mainly due to the lattice strain of the materials. This argument was further well supported with our XRD results. Also, it can be seen in Fig. 5 as the iron oxide concentration increases the amorphous carbon signal was suppressed due to the coverage of iron oxide particles on the surface.

### Catalytic activity

#### Role of activated carbon on adsorption/Fenton process

**Fig. 6:**
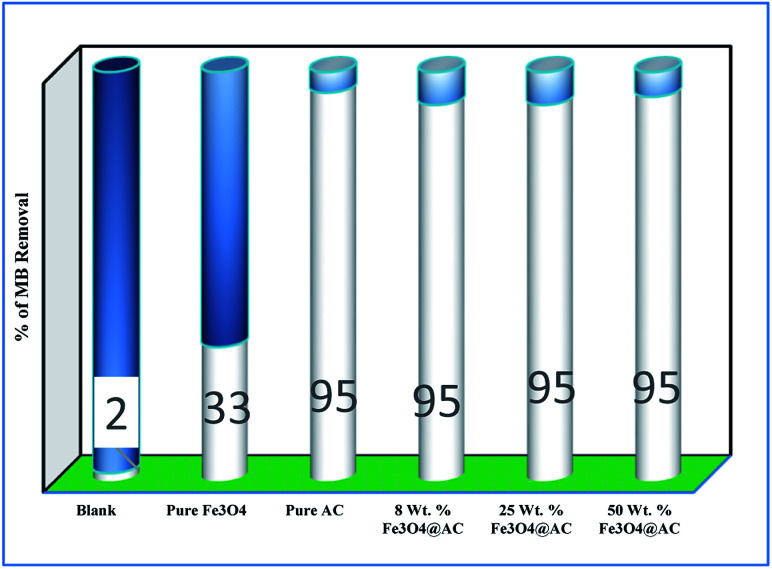
Effect of catalyst on adsorption and Fenton oxidation reaction of MB. Reaction conditions: initial concentration of MB: 4.48 × 10^−4^ M: H_2_O_2_ concentration 1.6 × 10^−3^ M: amount of catalyst: 7 mg medium: neutral pH: reaction time: 10 m; reaction temperature: 298 K. Stirring speed: 150 rpm.

**Fig. 7:**
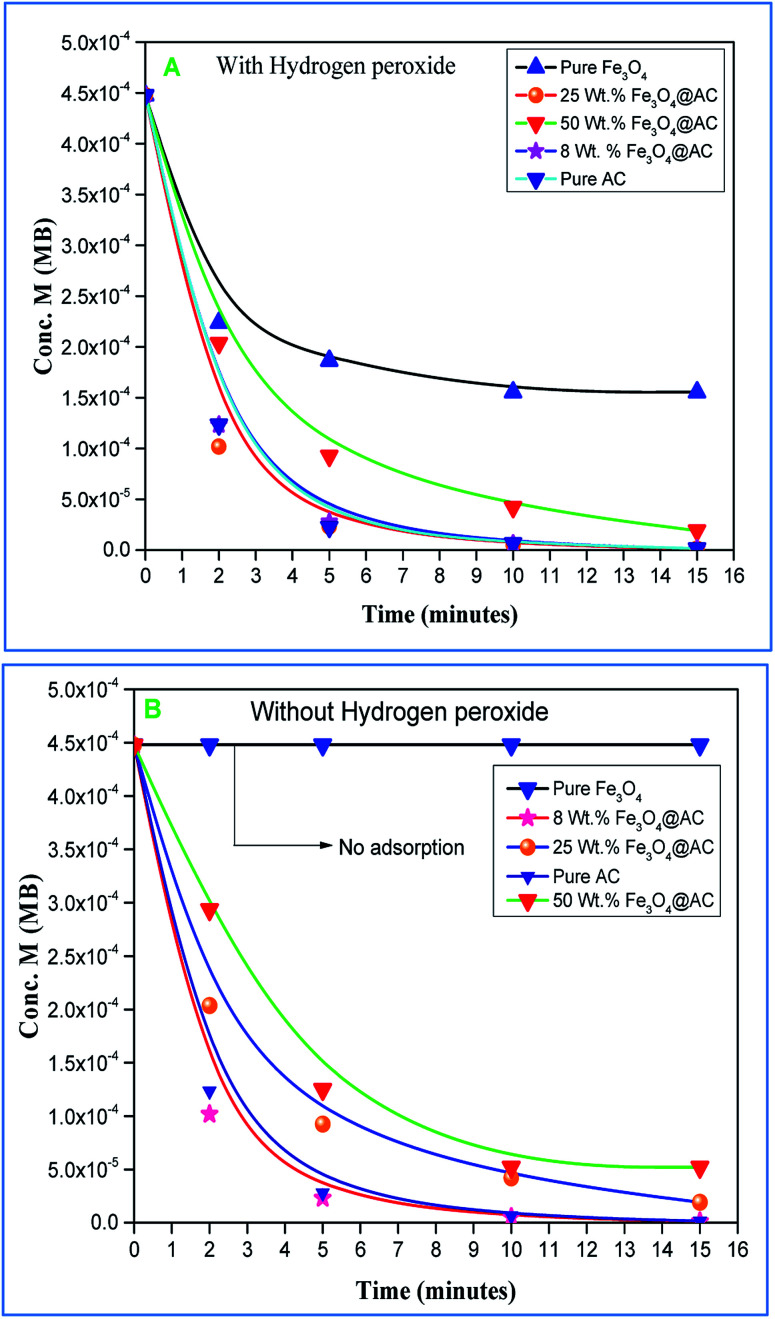
(A) (with H_2_O_2_) and (B) (without H_2_O_2_) adsorption study on synthesised catalysts. Reaction conditions: initial concentration of MB: 4.48 × 10^−4^ M: H_2_O_2_ concentration 1.6 × 10^−3^ M: amount of catalyst: 7 mg medium: neutral pH: reaction time: 10 m; reaction temperature: 298 K. Stirring speed: 150 rpm.

The Fenton oxidation reaction of methylene blue (4.48 × 10^−4^ M) was carried out over 8, 25 and 50 wt% of Fe_3_O_4_ loaded on activated carbon catalysts using diluted hydrogen peroxide solution at ambient reaction conditions, as shown in Fig. 6. All the reactions were performed in a batch reactor at the neutral pH medium. For comparison purposes, the Fenton's oxidation reaction was also performed with the help of pure Fe_3_O_4_ catalyst and the blank experiment (without any catalyst). The results are confirmed that the complete MB degradation was achieved on all the catalysts except pure Fe_3_O_4_. Unambiguously indicating that there was no significant difference between the pure and Fe_3_O_4_ decorated on activated carbon catalysts, it also clearly shows that the role of iron oxide was not clearly understood. This unusual trend is the most crucial part of the present investigation, and hence, the study was mainly focused on to understand the reaction pathway with a lot more physical significance. Unfortunately, this reaction involves many complicated steps (adsorption and radical/non-radical pathways), and hence it was difficult to conclude that reaction mainly proceeded either by Fenton's oxidation reaction or simple adsorption process. Hence, it is essential to minimize the many complicated steps at the early stage itself for better understanding of the role of catalyst. Initially, a blank test was performed with and without the addition of hydrogen peroxide solution, and we observed that Fenton's oxidation rate was almost negligible even after two weeks of induction time under identical experimental conditions (Fig. 6). These results are strongly emphasizing that the powerful OH radicals required for a complete MB oxidation reaction.^37^ The next experiment has performed with the help of pure Fe_3_O_4_ catalyst in the presence of hydrogen peroxide solution, and the obtained results are showing that the catalyst exhibited low catalytic activity, as compared to carbon-based catalysts. It specifies that the catalyst may possess a low surface area, or the catalyst might be deactivated quickly owing to extended leaching of either Fe^2+^ or Fe^3+^ ions to the reaction mixture and subsequently suppress the reaction rate at the initial stage itself.^38^ To investigate further the possible reason for low activity of pure Fe_3_O_4_, and execution of similar activity on all activated carbon-based catalysts, a time on stream study was studied, and the results were depicted in Fig. 7A and B. A blank experiment was also performed (without hydrogen peroxide) on all the catalysts to differentiate from physical adsorption (Langmuir adsorption isotherm) and Fenton's oxidation reaction. Predictably, the results are concluded that without hydrogen peroxide, the adsorption of methylene was found to be nearly 100% after 5 minutes of stirring time on carbon-based catalysts (the amount of adsorbed MB Conc. was provided in the Fig. 7B). This trend undoubtedly indicates that it follows the simple Langmuir adsorption isotherm rather than Fenton's oxidation reaction; however, this behaviour was not seen in the case of pure Fe_3_O_4_ catalyst due to the lack of high surface area of the material as compared to the activated carbon catalyst. It was interesting to find that, except pure Fe_3_O_4_ catalyst, all other catalysts are exhibited excellent adsorption capacity. Despite this adsorption phenomenon, the role of hydrogen peroxide and iron oxide active sites are not clear, and hence, we performed the additional test in the presence of added hydrogen peroxide to evaluate any significant improvement in the reaction rate for all the catalysts. Notably, the reaction profile clearly shows that there was no significant difference between these two experiments except Fe_3_O_4_ catalyst. This opposite trend on pure Fe_3_O_4_ catalyst unanimously confirms that it is well responded to the Fenton's oxidation reaction rather than by a simple adsorption process.

**Fig. 8:**
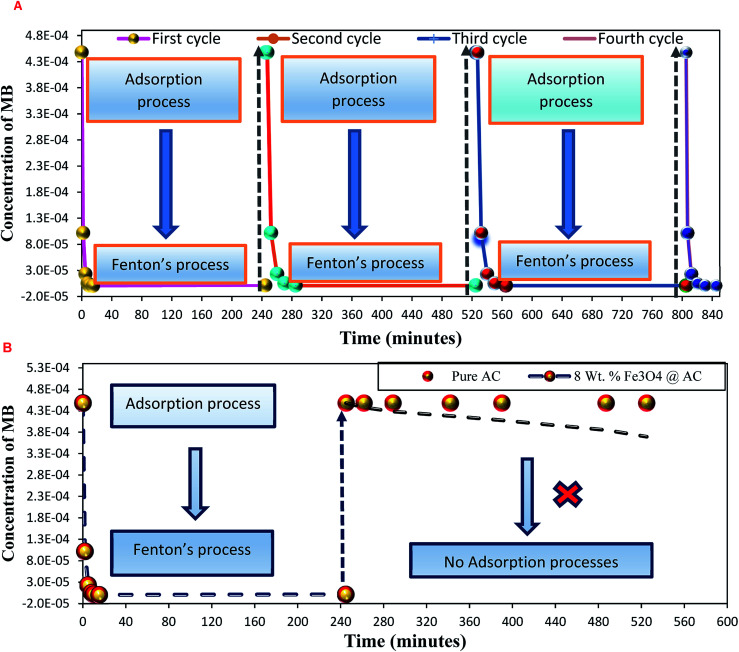
Effect of hydrogen peroxide on methylene adsorption study: (A) 25 wt% Fe_3_O_4_/activated carbon catalyst and (B) pure activated carbon and 8 wt% Fe_3_O_4_/activated carbon catalysts. Reaction conditions: H_2_O_2_ concentration 1.6 × 10 ^−3^ M: amount of catalyst: 7 mg; neutral pH: reaction temperature: 298 K; stirring speed: 150 rpm.

**Fig. 9:**
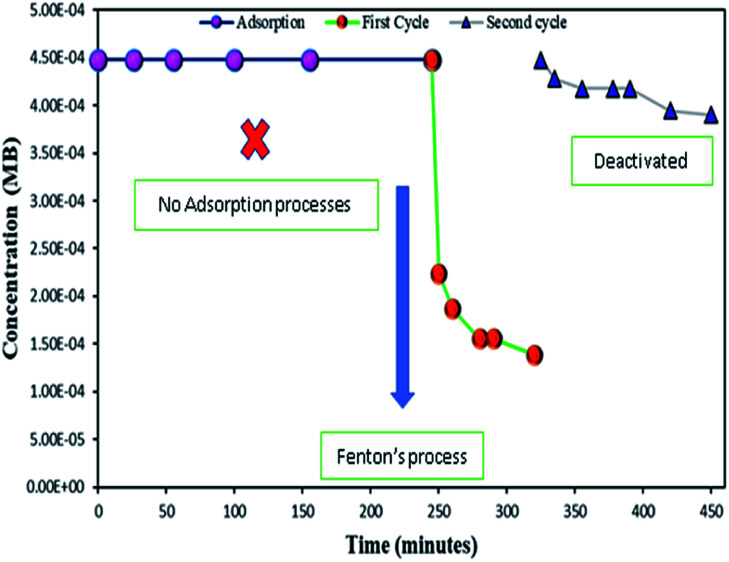
Effect of hydrogen peroxide on adsorbed methylene blue on activated carbon. Reaction conditions: H_2_O_2_ concentration 1.6 × 10^−3^ M: amount of catalyst: 7 mg; catalyst: pure Fe_3_O_4_; neutral pH: reaction temperature: 298 K; stirring speed: 150 rpm.

As mentioned above, the combined experimental results have proved that the adsorption rate was much faster than the oxidation reaction rate on all the catalysts (*i.e.*, 8, 25 and 50 wt% Fe_3_O_4_@AC). Hence, it was difficult to distinguish from adsorption reaction to oxidation reaction. Therefore, it is essential to investigate the role of hydrogen peroxide on iron oxide loaded on activated carbon catalysts for advanced Fenton oxidation reaction system. In fact, the main objective of the present investigation is to remove the organic pollutant from the industrially polluted water by a complete oxidation reaction pathway, but not merely by simple adsorption of methylene blue. Hence a systematic separate reaction study is required for better understanding of the Fenton oxidation reaction, which is a crucial part of the present investigation. Based on our preliminary analysis, the influence of hydrogen peroxide on MB degradation reaction was performed separately over iron oxide loaded on carbon catalysts, and the hydrogen peroxide concentration was kept as 1.6 × 10^−3^ M. The reaction conditions were shown in Fig. 8A and B. Initially, the methylene blue was allowed to adsorb on a 25 wt% Fe_3_O_4_@AC catalyst (optimized wt%) and forced to attain the complete equilibrium under the identical experimental conditions. The maximum up taken of methylene blue was found to be 359.9 mg g^−1^ at 30 °C in just 5 minutes of reaction time, which is very close to the maximum adsorptivity value (373.9 mg g^−1^), as reported by Aoumria Ouldmoumna *et al.*^39^ The adsorbed methylene blue on the carbon catalyst was further treated with hydrogen peroxide solution, and the reaction continued for another four hours to completely oxidize the adsorbed methylene blue by Fenton's oxidation process. After the oxidation treatment, the methylene blue concentration was increased to the initial value, and the adsorption study was continued on the same catalyst in the same solution, this trend shows that the adsorption behaviour was very similar to the initial run (Fig. 8A). It is a clear indication of all the adsorbed methylene blue can be effectively oxidized by hydrogen peroxide and subsequently clean the surface of activated carbon materials and further help to reabsorb the methylene blue molecule. Further, we spike the methylene blue concentration to initial concentration, and we allowed to attain the adsorption equilibrium and continued the Fenton oxidation reaction. The same methodology was adopted for three consecutive cycles, and we observed that there was no significant difference from the first cycle to the fourth cycle. This cyclization process is very much similar to that of an activation step as used in water purification technology. Surprisingly, the above-mentioned trend was not seen on 8 wt% Fe_3_O_4_ loaded on activated carbon catalyst, in fact, the activity was stopped even in the second cycle of the reaction itself (Fig. 8B). It demonstrates that the optimum amount of Fe_3_O_4_ species is required on the surface of the catalyst to produce the powerful OH radicals to further attack the adsorbed MB molecule on the catalyst sites. For comparison purpose the same methodology was employed on pure activated carbon catalyst, and it was observed that there was no oxidation ability seen even in the presence of high concentrated hydrogen peroxide solution on adsorbed MB activated carbon material (Fig. 8B). Hence, the study proved that either Fe^3+^ or Fe^2+^ ions are required for advance Fenton oxidation process. Also, we observed that the adsorption rate is very much similar to all the catalysts, including the pure activated carbon material. The results clearly explain that iron oxide particles did not play any significant role in improving the adsorption capacity. Additionally, the pure Fe_3_O_4_ activity was also investigated by using a similar strategy for meaningful comparison, and the results were depicted in Fig. 9. As we noted that the MB concentration did not drop even after 100 minutes of reaction time, manifesting that there was no physical adsorption of MB taking place over the catalyst, possibly it might be due to the low surface area nature of the material. On the other hand, it shows better activity in the presence of hydrogen peroxide due to the formation of hydroxyl radicals through the Fenton process, but the activity was not retained in the second cycle of reaction due to deactivation of the catalyst either by strong adsorption of MB or complete oxidation of Fe^2+^ ions into Fe^3+^.^38^ Based on the information we gained from the above experimental results, the possible reaction mechanism can be exploited; however, this reaction was believed to proceed *via* several complicated radical reaction pathways, and hence, few more additional experiments are required to propose the meaningful reaction mechanism for methylene blue degradation reaction. Most importantly, the identification of the real role of iron oxide species for the methylene blue Fenton oxidation reaction on the surface of the activated carbon catalyst is a crucial part of this work and hence, a systematic analysis was carried out with the help of several homogeneous Fenton oxidation reactions with and without added radical scavengers. Initially, the ferrous sulphate solution (0.005 M) was treated with the known concentration of hydrogen peroxide solution (1.6 × 10^−3^ M), as optimized from the above experimental results, and the results proved that as soon as the addition of Fe^2+^ in peroxide solution, it would interact with hydrogen peroxide molecule and produce the powerful OH radicals. The resultant OH radicals may subsequently combine with different types of hydroxy/hyrodxyperoxo radicals and produced the oxygen gas with the evolution rate of 1.6 mL min^−1^ g_cat_^−1^ along with the formation of orange-yellow colour precipitation at the end of the reaction.

**Scheme 1:**
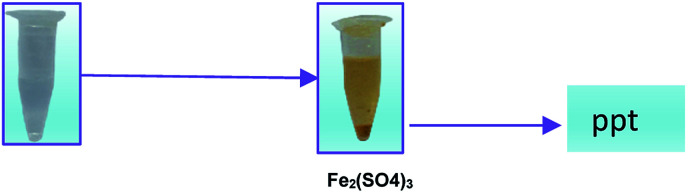
The formation of Fe(OH)_3_ precipitate from Fe^2+^ ion solution.

This also confirms that the OH anions would be produced and it reacts with Fe^3+^ ion to form an insoluble Fe(OH)_3_ species. Thus, it proves the reaction mainly occurred based on the classical Fenton equation, as shown in Scheme 1.

i Fe^2+^ + H_2_O_2_ → Fe^3+^ + OH˙ + OH^−^

ii
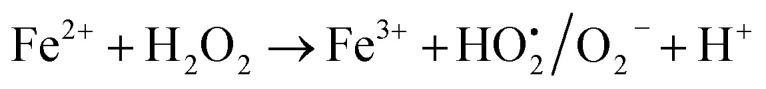
iii
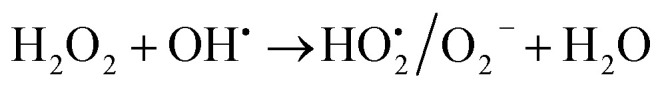
iv
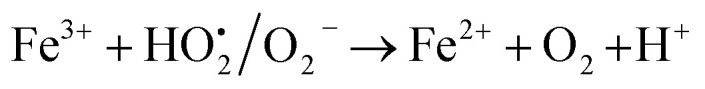
Overall reaction;

13 Fe^2+^ + H_2_O_2_ → Fe(OH)_3_ ppt + O_2_

To further confirm the formation of OH radicals, the powerful radical scavenger (acetone) was added in the solution,^40^ and we observed that there was no oxygen gas was evolved, but it forms the iron hydroxide precipitate in the solution indicates that the all radicals completely were trapped by radical scavengers (as shown in eqn (14)). This analytical method proves that the reaction mainly occurred only by the OH radical formation pathway, as reported by many researchers.

14 Fe^2+^ + H_2_O_2_ + acetone (radical scavanger) → Fe(OH)_3_ ppt + No O_2_

**Fig. 10:**
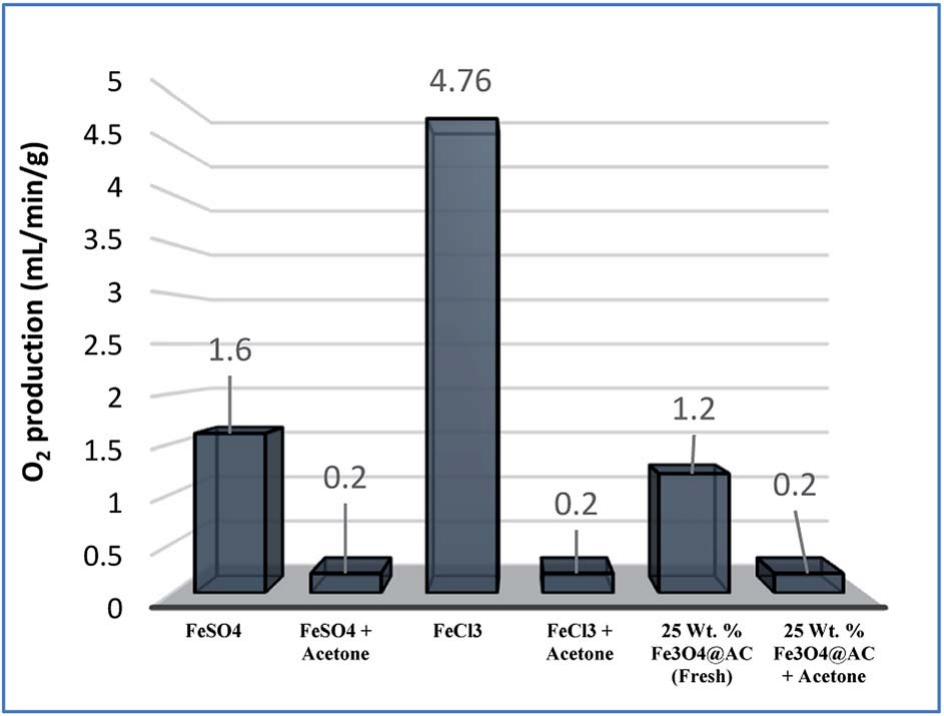
Effect of radical scavengers on various hetero and homogenous catalyst. Reaction conditions: Conc. of FeSO_4_ – 0.05 M; Conc. of FeCl_3_ – 0.05 M; ratio of radical scavenger to catalyst (1 : 4); Conc. of H_2_O_2_; 0.125 M; amount of solid catalyst: 7 mg; reaction time: 30 m; stirring speed; 150 rpm.

**Scheme 2:**
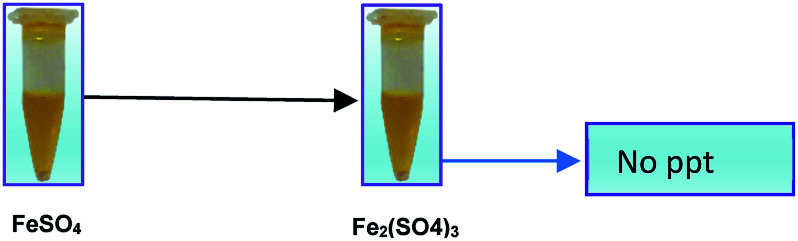
The absence of Fe(OH)_3_ precipitate from Fe^3+^ ion solution.

A similar reaction was performed with Fe^3+^ solution (ferric chloride) to infer any significant difference between Fe^2+^ and Fe^3+^ ions. Since the catalyst, we prepared eventually contains both the oxidation states, as evident from XRD analysis. It can be seen from Fig. 10 that the rate of production of oxygen was significantly higher than the previous experiment, and remarkably there was no evidence for the formation of Fe(OH)_3_ species (Scheme 2, according to eqn (15) and (16)), indicating that the reaction was mainly initiated *via* OOH radical pathway, according to eqn (V)–(XI). Due to the absence of iron hydroxide precipitate formation, the reaction did not proceed *via* the elementary step of (VII) and (IX) and hence, the rate constant values have not been included in the overall rate consideration. After the addition of radical scavenger, the oxygen evolution reaction rate decreased from 5 mL min^−1^ to 0.2 mL min^−1^, it confirms that the reaction mainly executed *via* the OOH pathway without the formation of OH anions, also, it proves that the reaction not proceeded by barely OH radicals alone. In addition to that, we observed the acetone could act as a good scavenging activity to trap both OH and OOH radicals, respectively.V
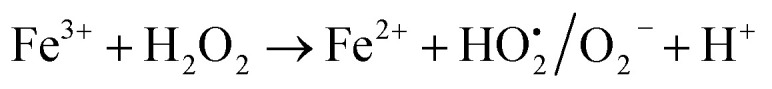
VI
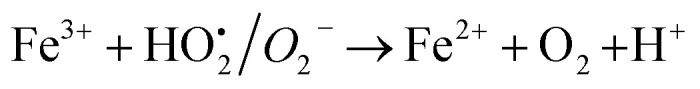

VII Fe^2+^ + OH˙ → Fe^3+^ + OH^−^

VIII
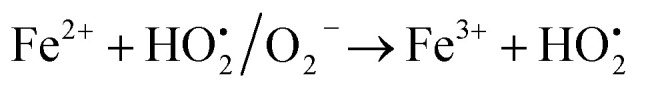

IX OH˙ + OH˙ → H_2_O_2_

X
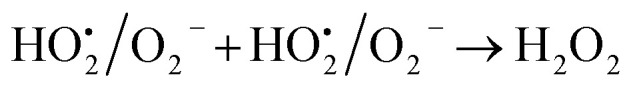
XI
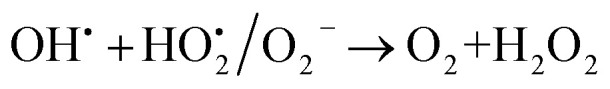

15 Fe^3+^ + H_2_O_2_ → No Fe(OH)_3_ ppt + oxygen was evolved

Overall reaction;

16 Fe^3+^ + H_2_O_2_ + acetone (radical scavanger) → No Fe(OH)_3_ ppt + No O_2_

For comparison, the MB degradation reaction was also performed in the presence of MB solution and, it was found that in both the experiments the decolorization activity was observed; however, their reaction rates were not the same. The difference in activity was mainly attributed due to the formation of two different types of radicals with two different rate constants, as depicted in eqn (13). In general, OH radicals are more reactive than the OOH radicals, which are directly formed due to the interaction of peroxide molecule with Fe^2+^ and Fe^3+^ sites, respectively. However, many researchers did not account for the OOH radicals into degradation efficiency because the rate of formation of hydroperoxide radicals is relatively lower than the hydroxyl radical formation rate. Nevertheless, this tendency was contrary to the oxygen evolution reaction there we observed that the oxygen production rate from Fe^2+^ sites was significantly larger than one which arises from the Fe^2+^ ion sites. The difference in rate might be due to dominating the recombination effect of hydroperoxyl radicals, which subsequently enhances the rate of oxygen production and thereby decreases the MB degradation rate. Hence, the overall homogenous Fenton's study concluded that both types of radicals are effectively participating in the MB degradation under identical experimental conditions, but the rate of degradation was not similar due to variable types of radicals are produced in the solution.

In the Recent past, Rachmilovich-Calis *et al.*^41^ proposed the degradation reaction mechanism for various types of dye molecules and, they concluded that the reaction mainly occurred not only due to the formation of OH radicals but also due to the involvement of Fe–OOH species in the reaction mixture. These results are inconsistent with our homogenous catalytic study. Based on the experimental results, the complete degradation reaction was mainly occurred due to the involvement of both Fe^2+^ and Fe^3+^ ions in the interface reactions. The same methodology was employed over 25 wt% Fe_3_O_4_@AC catalyst to estimate the real active sites for MB degradation reaction. Initially, the Fenton's oxidation reaction was performed in the presence of only hydrogen peroxide solution, and the oxygen production rate was calculated. The obtained results are illustrated in Fig. 10. In the first cycle of reaction, the rate of formation of oxygen was found to be 1.2 mL min^−1^; however, the oxygen production rate has diminished in the next consecutive cycles.

A possible reason can be arrived based on the existence of loosely bounded Fe^3+^ ion species in the activated carbon matrix, and that could be responsible for oxygen evolution reaction. In the second cycle of reaction, the catalyst was separated from the reaction mixture and washed with distilled water several times, and the reaction was continued with freshly prepared dilute hydrogen peroxide. The observed results are showing that there was no evidence for the oxygen evolution reaction, as we expected from the initial run. These results are inconsistent with our recyclable experiment, as shown in Fig. 12.

**Fig. 11:**
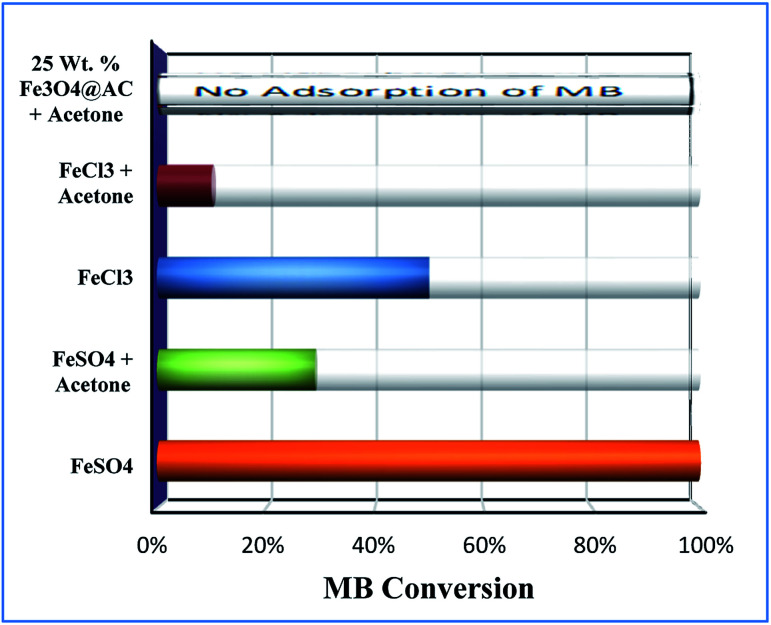
Effect of radical scavengers on MB degradation. Reaction conditions: Conc. of FeSO_4_ – 0.005 M; Conc. of FeCl_3_ – 0.005 M; ratio of radical scavenger to catalyst (1 : 4); Conc. of H_2_O_2_; 1.6 × 10^−3^ M; Conc. of MB 2.0 × 10^−4^ M; amount of catalyst: 7 mg; reaction time: 30 m; stirring speed: 150 rpm.

Furthermore, the adsorption efficiency of MB on the washed catalyst completely diminished after the addition of radical scavenger in the solution, rationalize that radicals are mainly generated on the surface rather than from the solution containing Fe^2+^/Fe^3+^ ions. Hence, the overall study proves that the Fenton's oxidation reaction is facilitated by both Fe^2+^ and Fe^3+^ ions with a lesser amount of oxygen evolution reaction (Fig. 11).

### Deactivation study

**Fig. 12:**
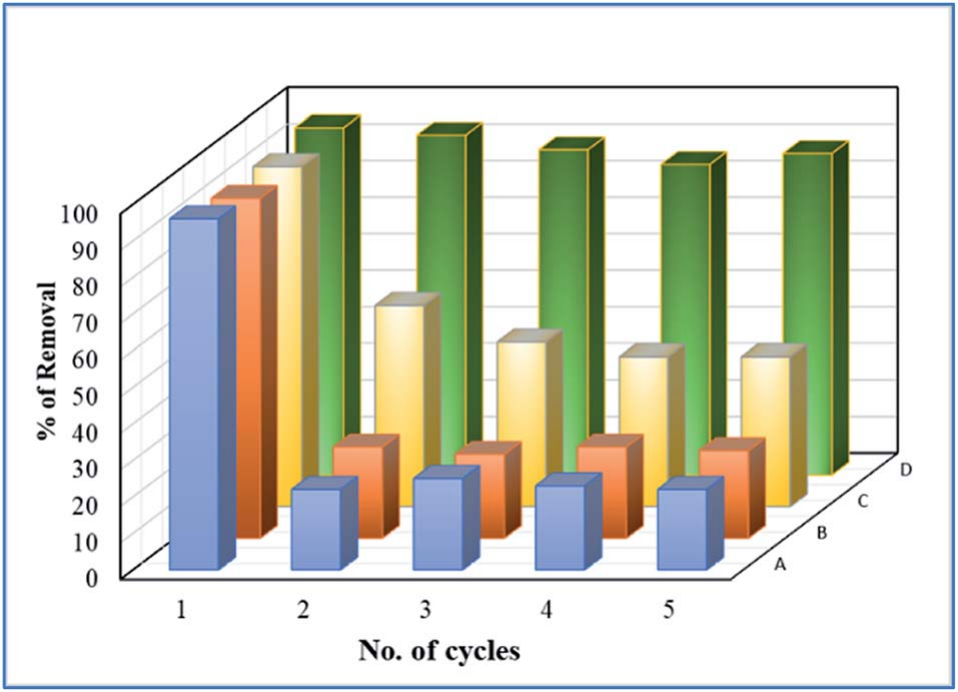
Deactivation study. Reaction conditions: Initial concentration of MB: 4.48 × 10^−4^ M: H_2_O_2_ concentration 1.6 × 10^−3^ M: amount of catalyst: 7 mg medium: neutral pH: reaction time: 10 m; reaction temperature: 298 K. Stirring speed: 150 rpm. Catalysts (A) pure Fe_3_O_4_; (B) 8 wt% Fe_3_O_4_@AC; (C) 50 wt% Fe_3_O_4_@AC; (D) 25 wt% Fe_3_O_4_@AC.

To study the sustainability and reusability of the catalyst, the spent catalyst was used for the second set of a cycle without any additional treatment (Fig. 12). The 25 wt% loaded on the catalyst retained almost 85% of their initial activity and the results are completely in contrast to the pure Fe_3_O_4_ catalyst. The initial high catalytic activity was mainly due to the extended leaching of Fe^2+^/Fe^3+^ species during the course of reaction. However, in the second cycle, the reaction rate completely diminished due to the total oxidation of Fe^2+^ ion into Fe^3+^ on the catalyst matrix. The leached iron species was identified by treating the reaction mixture in aqueous ammonia solution, and it produced brown iron hydroxide precipitate at the bottom of the reaction mixture. The overall observation was concluded that 25 wt% Fe_3_O_4_@AC catalyst is a promising catalyst for cleaning the dye contaminated water in an inexpensive route.

### Effect of strain on catalytic activity

In general, the function of a catalyst is describing that the change of rate (and correspondingly, selectivity) reaction without affecting the catalyst structure. Most commonly, the reaction has proceeded with several elementary steps. During each elementary step in a heterogeneous catalyst system, the initial state (IS) transferred to the transition state (TS) on the surface of the catalyst. However, the catalyst does not control the competitive adsorption from one adsorbate relative to another, since the adsorption strength was differing from one intermediate to another is known as scaling relations.^42^ A more reactive catalyst surface is expected to bind all adsorbates (initial state, transition state, and final state) strongly than the non-reactive surface. Despite the scaling relation provide much information about the sites, but it has its limitations due to the marginal changes in the IS and TS as reported elsewhere. Due to its limitation in the catalyst field, the attempts have been (theoretically and experimentally) made to break the scaling relations (For instance creating second active sites, and creating co-ordination unsaturated sites, *etc.*).^43,44^ In another way, the reactivity of catalyst surface can be increased by creating a more straining environment, consult ref,^45–47^ and citations therein. A surface strain can be created either by applying mechanical loading or creating more defects (dislocations/imperfections) on the catalyst.^48^

**Fig. 13:**
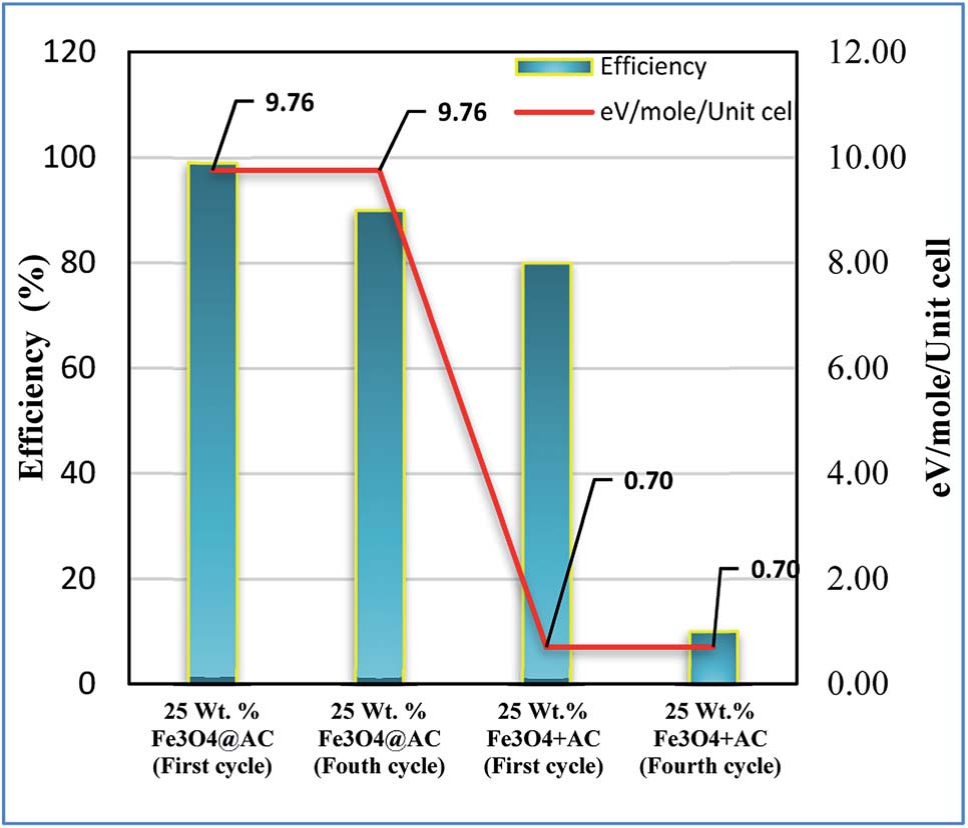
Influence of strain energy on Fenton's oxidation reaction; Reaction conditions: initial concentration of MB: 4.48 × 10^−4^ M: H_2_O_2_ concentration 1.6 × 10^−3^ M: amount of catalyst: 7 mg medium: neutral pH: reaction time: 10 m; reaction temperature: 298 K. Stirring speed: 150 rpm (physically mixed catalyst designated as 25 wt% Fe_3_O_4_ + AC).

Based on the information gained from the literature, we are trying to correlate the geometrical effect of the catalyst with catalytic activity. To optimize the geometrical factor of the catalyst which directly influence the Fenton oxidation reaction, the pure Fe_3_O_4_ was physically mixed with activated carbon to achieve the 25 wt% composition and results were compared with the 25 wt% Fe_3_O_4_@AC catalyst. The physically mixed catalyst activity was dropped significantly in the second cycle of reaction proves that the iron oxide material does not tolerate in the aqueous solution. The difference in activity was directly compared with our W–H derived model. For comparison, the anisotropic nature of the crystal was considered on both the catalysts to evaluate the strain effect influencing the catalytic activity. From the W–H model calculations, we confirmed that the 25 wt% Fe_3_O_4_@AC catalyst possesses higher lattice strain (along the *y* and *z*-axis), which facilitates to reduce the energy barrier between the initial state and final state, respectively. On the other hand, a surface strain creates many defective sites on the chemically mixed catalyst, and it helps to break the MB bonds easily by OH radicals on the surface. As shown in Fig. 13, unlike chemically mixed catalyst, the physically mixed was deactivated quickly either due to extended leaching of Fe^2+^ ion in the solution or the oxidation of Fe^2+^ to Fe^3+^. In order to investigate the extent of meal ion leaching from the catalyst after the third cycle of experiment, the ICP-OES analysis was performed. The detected amount of iron present in the solution was summarized in Table 3. In the previous report concluded that the deformation energy was broadly classified into two types based on the eV per atom variations (>100 meV is stronger; < 50 meV is weak deformation energy) as predicted from the DFT calculations.^49^ These results are in good agreement with our calculated model. The chemically interacted (25 wt% Fe_3_O_4_@AC) catalyst possesses high deformation energy (9.76 eV per mol per unitcell) than the other catalysts. As a result, the bond arrangements are relatively stronger on 25 wt% Fe_3_O_4_@AC material than weakly deformed catalysts; consequently, the metal ion has not been easily leached out from the lattice sites (Table 3). Further, the deactivation profile proves that the iron oxide particle in the chemically mixed catalyst was in more confinement environment, therefore, the strain, and deformation energy dramatically increased. The resultant steric confinement effect may prevent the iron oxide leaching as well as the surface oxidation of Fe^2+^ into Fe^3+^ ion. The results are concluding that the use of activated carbon support creates a large number of coordinatively unsaturated sites in a more strain environment and to maintain the heterogeneity of catalyst for the continuous Fenton's oxidation reaction.

**Table 3:** Correlation of activity *vs.* lattice strain

Catalysts	Lattice strain *ε* (10^−3^)	Deformation energy eV per mol per unitcell	MB Conv. (%)	The amount of leached iron^a^ (mg L^−1^)
Pure Fe_3_O_4_	0.92	0.70	22	35
25 wt% Fe_3_O_4_@AC	3.43	9.76	88	7.3
50 wt% Fe_3_O_4_@AC	2.19	3.98	41	35

a Determined from ICP-OES analysis.

### Possible reaction pathway for adsorption/Fenton process

**Fig. 14:**
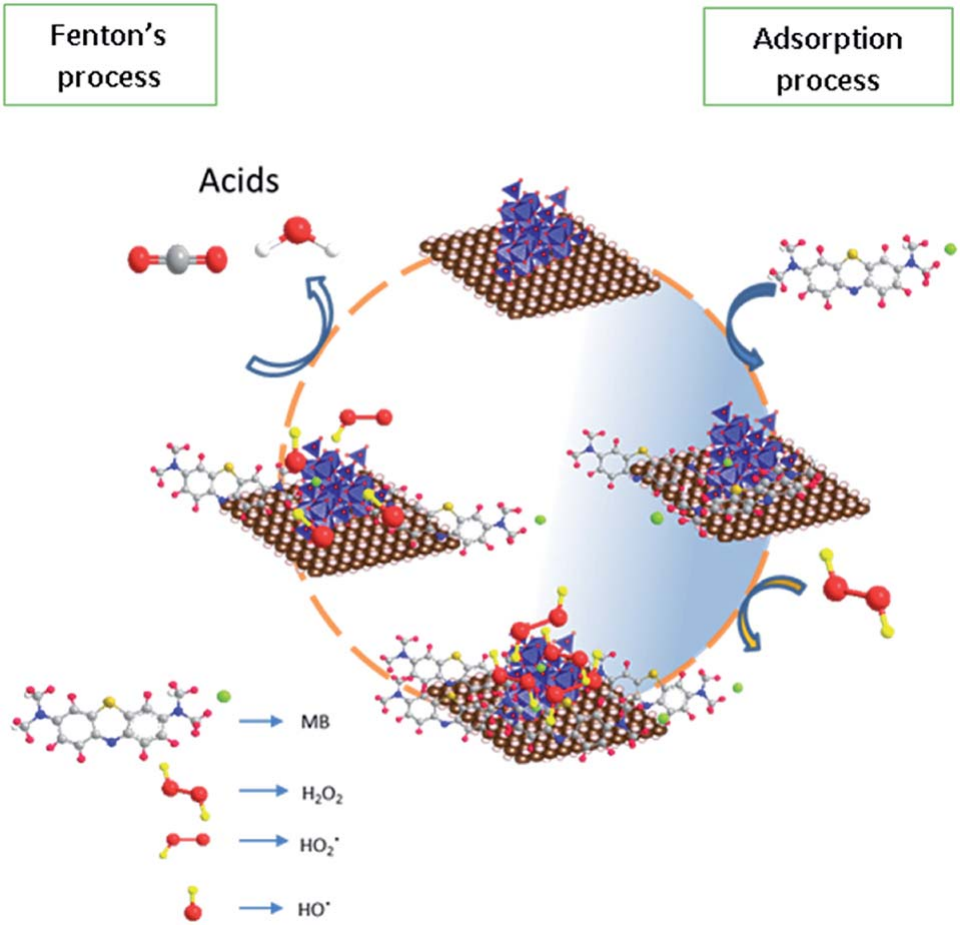
Proposed reaction mechanism for adsorption and Fenton's oxidation reaction.

Based on the reaction trend, a possible reaction mechanism for the oxidation of methylene blue on the surface was proposed in Fig. 14. In the first step, the methylene blue was allowed to occupy the sites completely according to Langmuir adsorption isotherm and then treated with hydrogen peroxide to generate the hydroxyl radicals *via* a dissociative pathway on the surface of defective iron oxide sites.

**Fig. 15:**
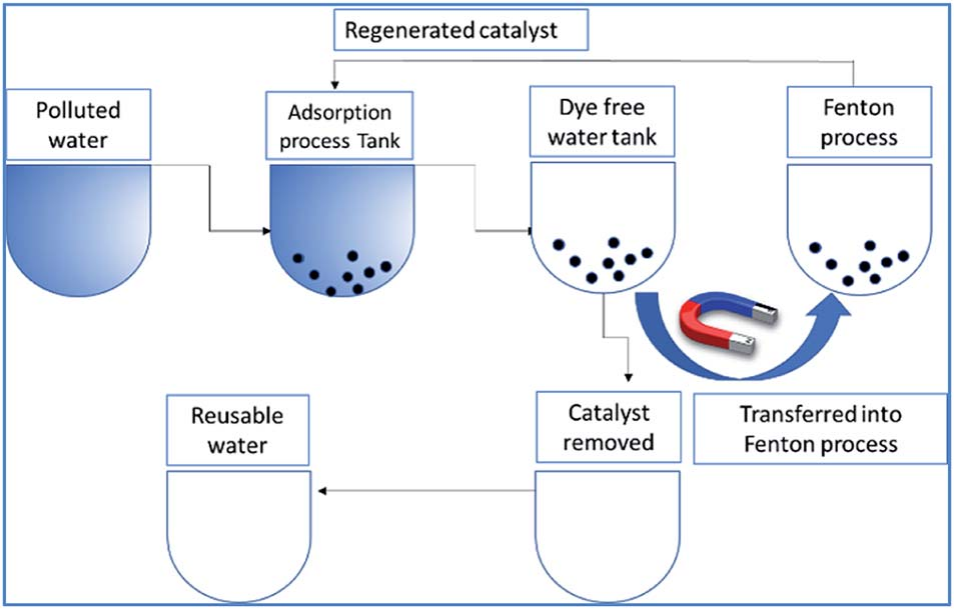
Process flow diagram of the proposed technology.

The electron transfer reaction was initiated by Fe^2+^/Fe^3+^ ions and subsequently activated the hydrogen peroxide molecule. The resultant 
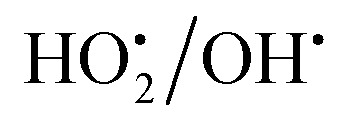
 radicals attack the Methylene blue molecule on the adsorbed surface, which subsequently converted into carbon dioxide and water. A detail process flow diagram was shown in Fig. 15.

## Conclusions

The Fe_3_O_4_ particles are embedded on activated carbon catalyst with different weight percentages (8, 25 and 50 wt%@AC) prepared by co-precipitation method. The embedded iron oxide particles were investigated by XRD, Raman and SEM analysis. The XRD and Raman analysis is supporting that iron oxide particle has located in a 25 wt% catalyst with a more strain environment. The lattice strain was developed due to the interaction of iron oxide in the carbon matrix, as investigated by using a modified W–H model. The results are confirmed that most of the iron oxide mainly located inside the pores of carbon material, and it is responsible for the higher uniform deformation energy density in the overall crystal system. The synthesized materials are evaluated for advanced Fenton's oxidation reaction. Among the catalysts, the 25 wt% loaded on carbon catalyst exhibited both excellent adsorption and Fenton's oxidation ability in the peroxide medium. The reusability tests carried out on all the catalysts, and we observed that 25 wt% Fe_3_O_4_@AC catalyst exhibited similar catalytic activity up to 4 consecutive cycles than the physically mixed catalyst. The study concluded that anisotropic strain effect of modified Fe_3_O_4_ (eV per mol per unitcell) was played an important role to maintain the heterogeneity of the catalyst. The given experimental results under the strain relations can provide a new possible pathway for the future design of heterogeneous catalysts for longtime durability for many reactions. Further, the combined adsorption/Fenton's strategy provided a new insight for the removal of dye stuff from the industrially polluted wastewater.

## Experimental

The synthesis procedure and characterization techniques used in this study were provided in the ESI (Section 2.2 and 2.3).†

### Catalytic activity (adsorption/Fenton's process)

**Fig. 16:**
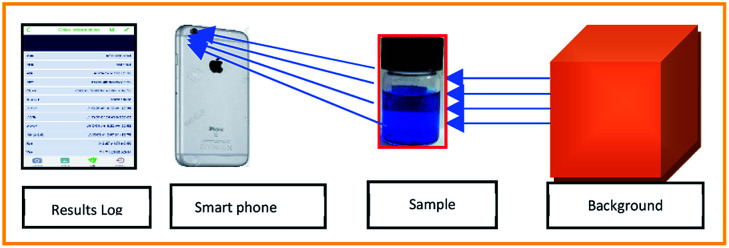
Method of analysis of Conc. of MB using smart phone technology.

The adsorption study was performed in a 5 mL micro-glass reactor containing 2.5 mL of water contaminated with a known concentration of MB. Typically, 7 mg of catalyst was dispersed uniformly in the solution and the pH of the solution was found to be around 5 in the reaction mixture. After the adsorption period, a required concentration of H_2_O_2_ added under constant stirring rate. The concentration of MB during the reaction was monitored with the help of the colour analyser application (version 2.0) using the iPhone 6 s smartphone, as shown in Fig. 16.

The exact concentration was predicted from the constructed calibration curve, as shown in Fig. S1.† The absorbance was calculated from the Beer's law equation (*A* = −log(*I*_o_/*I*). The curve exhibited perfect linearity without any significant deviation from the regression coefficient value. The use of smartphone technology will be enabled to identify the unknown concentration of methylene blue rapidly; in fact, it does not require any expensive spectrophotometer analysis. The results were perfectly correlated with the UV analysis within 10% of experimental errors. To confirm the decolorization efficiency, the reaction mixture was further analyzed by both UV and HPLC instruments (see Fig. S3†).

## Conflicts of interest

There are no conflicts to declare.

## Supplementary Material

RA-010-D0RA07866B-s001
