# Supplementary material for: Influence of lattice strain on Fe3O4@carbon catalyst for the destruction of organic dye in polluted water using a combined adsorption and Fenton process
Source: RSC Adv. 2020 Oct 26;10(64):39146–59. doi: 10.1039/d0ra07866b (PMC9057358; doi:10.1039/d0ra07866b)
Supplement: RA-010-D0RA07866B-s001 [file RA-010-D0RA07866B-s001.pdf]

Electronic Supplementary Material (ESI) for RSC Advances.
This journal is © The Royal Society of Chemistry 2020

Electronic supplementary information for

Influence of lattice strain on Fe_3O_4@carbon catalyst for the destruction of organic dye in polluted water using combined adsorption and Fenton's process

D. Santhanaraj[a*], N. Ricky Joseph[a], V. Ramkumar[b], A. Selvamani[c], I.P. Bincy[d], K. Rajakumar[e]

[a]Department of Chemistry,

Loyola college,

Chennai, 600 034,

Tamilnadu, India.

[b]Department of Polymer Science and Technology,

Council of Scientific and Industrial Research (CSIR) – Central Research Laboratory, Adyar,

Chennai 600 020,

Tamilnadu, India.

[c]Catalytic Reforming Area, Light Stock Processing Division,

CSIR- Indian Institute of Petroleum

Dehradun-248 005, Uttarakhand

[d]Department of Physics,

MES College,

Nedumkandam, Kerala 685 553, India.

[e]Nanotechnology Research & Education Centre

South Ural State University,

Chelyabinsk - 454080, Russia

*Corresponding author. +91 7092053305

E-mail address: santhanaraj@loyolacollege.edu

2. Experimental

2.2 Chemicals

All chemicals used in this study were analytical grade and used without any further purification methods. Ferrous sulphate ($FeSO_4$. $4H_2O$ SRL, India), Activated carbon (Merck, India), diluted HCl acid (Merck 35 %), Aqueous ammonium hydroxide (Aq.NH_3, 25 % Merck), Ferric chloride ($FeCl_3$ $6H_2O$), Methylene blue (SRL, India), Iron (III) oxide (Fe_2O_3, SRL), and hydrogen peroxide (H_2O_2, 30% SRL, India).

2.3 Synthesis of magnetic Fe_3O_4 particles

The required amount of 0.01 M $FeSO_4.4H_2O$ and 0.02 M $FeCl_3.6H_2O$ was dissolved in a 200 mL of degassed distilled water under constant stirring. [1] Aqueous ammonium hydroxide (NH_4OH) solution was added drop by drop to raise the pH of the solution to 8.0. At this pH, the solution turned from brown to black. Subsequently, the black particles were quickly washed three times with distilled water and dried at 100 °C in the air environment.

2.4 Synthesis of Fe_3O_4 particle on activated carbon (8 % and 25 % Fe_3O_4@AC)

The 8 and 25% of Fe_3O_4 was loaded on commercially available activated carbon by precipitation method.[2] Initially, the calculated amount of activated carbon (0.46, 0.38 and 0.25 g was used to achieve 8, 25, and 50 Wt.% Fe_3O_4@AC catalysts) was dispersed on distilled water by a mechanical stirring method. Then, the required amount of $FeSO_4$ (0.048, 0.15 and 0.3 g was used to prepare for 8, 25, and 50 Wt.% Fe_3O_4@AC catalysts) in HCl acid was added in the solution and the stirring was continued for another two hours in the nitrogen environment. Aqueous ammonium hydroxide (NH_4OH) solution was added drop by drop to raise the final pH of the solution to 8.0. At this pH, the solution was turned from brown to black. Subsequently, the black particles were quickly washed three times with distilled water and dried at 100 °C in the air environment. The synthesised materials are exhibited well magnetic property. The advantage of using a magnetic particle as a catalyst in this study can easily separable from the reaction mixture with the help of a simple external permeant magnet at the end of the reaction (Fig. S1).

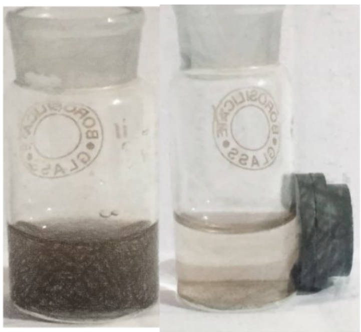

Fig. S1: Effect of external magnetic field on magnetic Fe_3O_4@AC particles

2.5 Characterization of the catalysts

The synthesized catalyst phase formation was verified using the PANalytical Xpert instrument, and the analysis was carried out at room temperature using Cu K-α radiation in the two-theta range of 10° to 80 °. The full-width half-maximum (FWHM) of each diffraction plane was obtained from the origin 9.0 software by using the Gaussian equation. The amount of iron present in the reaction mixture and the catalysts were determined by ICP-OES analysis (Perkin-Elmer OPTIMA 3000). The surface morphology and the location of iron oxide on the activated carbon catalysts were viewed by using scanning electron microscopy. Initially, the sample was placed on the stub and then coated with gold particles for 2 min using ion sputter coater and viewed on the HITACHI ESEM instrument. The FT-Raman spectra were recorded on a Thermo Fischer DXR, USA instrument equipped with He-Ne Laser with the excitation wavelength of 532 nm.

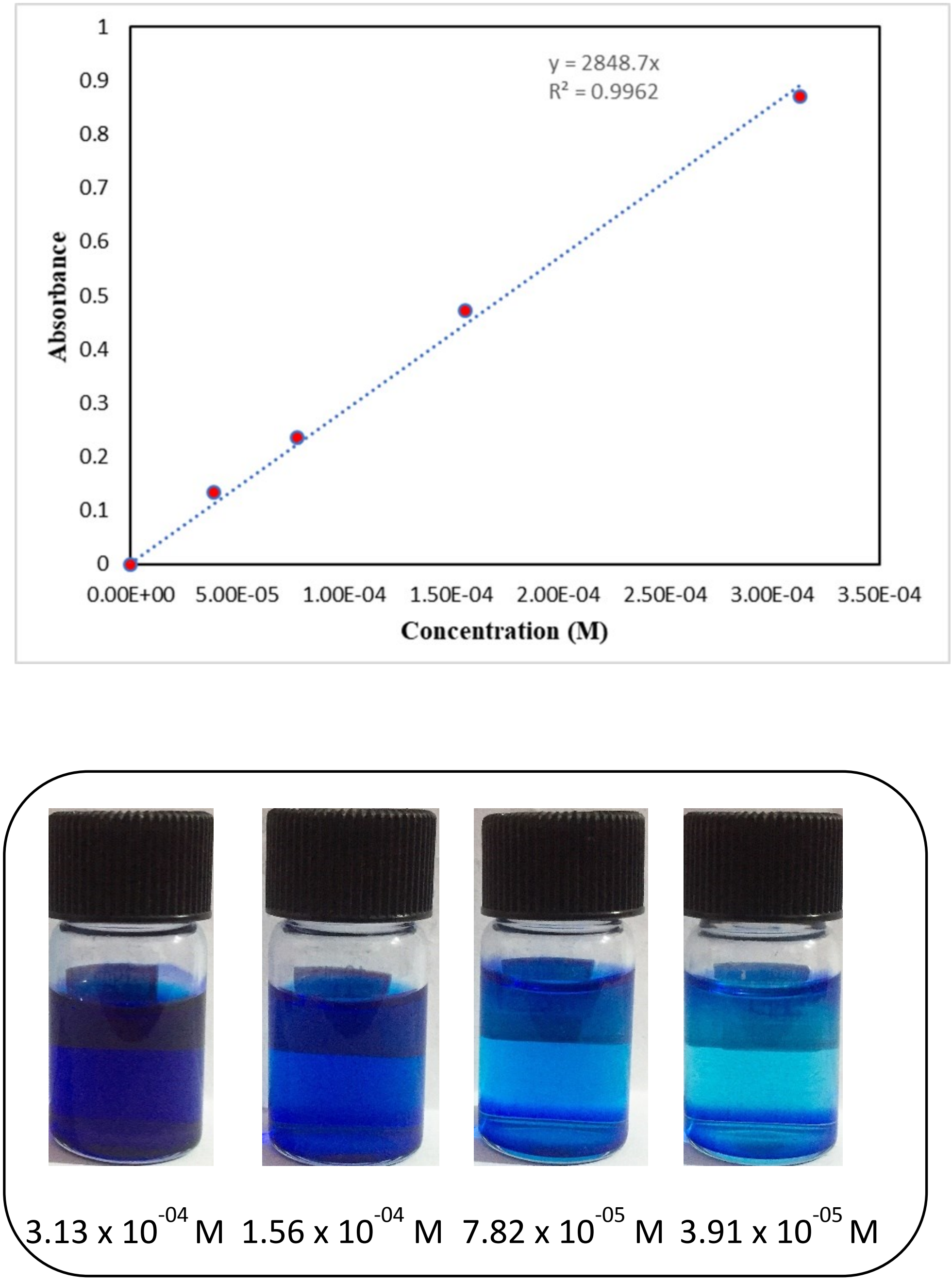

Fig. S2: Calibration curve for MB obtained from smart phone colour analyser application.

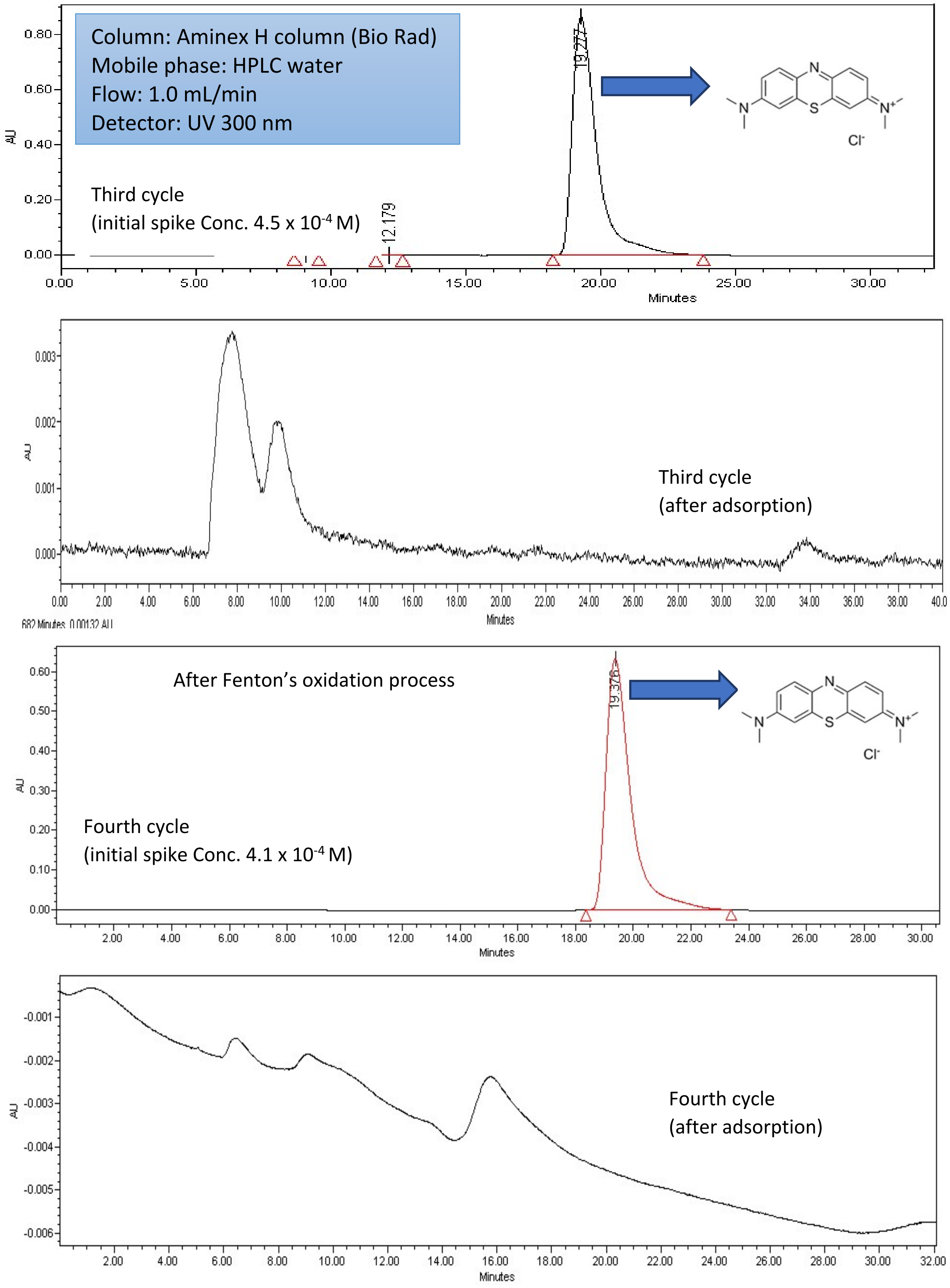

Fig. S3 HPLC Chromatogram of third and fourth cycle of adsorption/Fenton's process sample

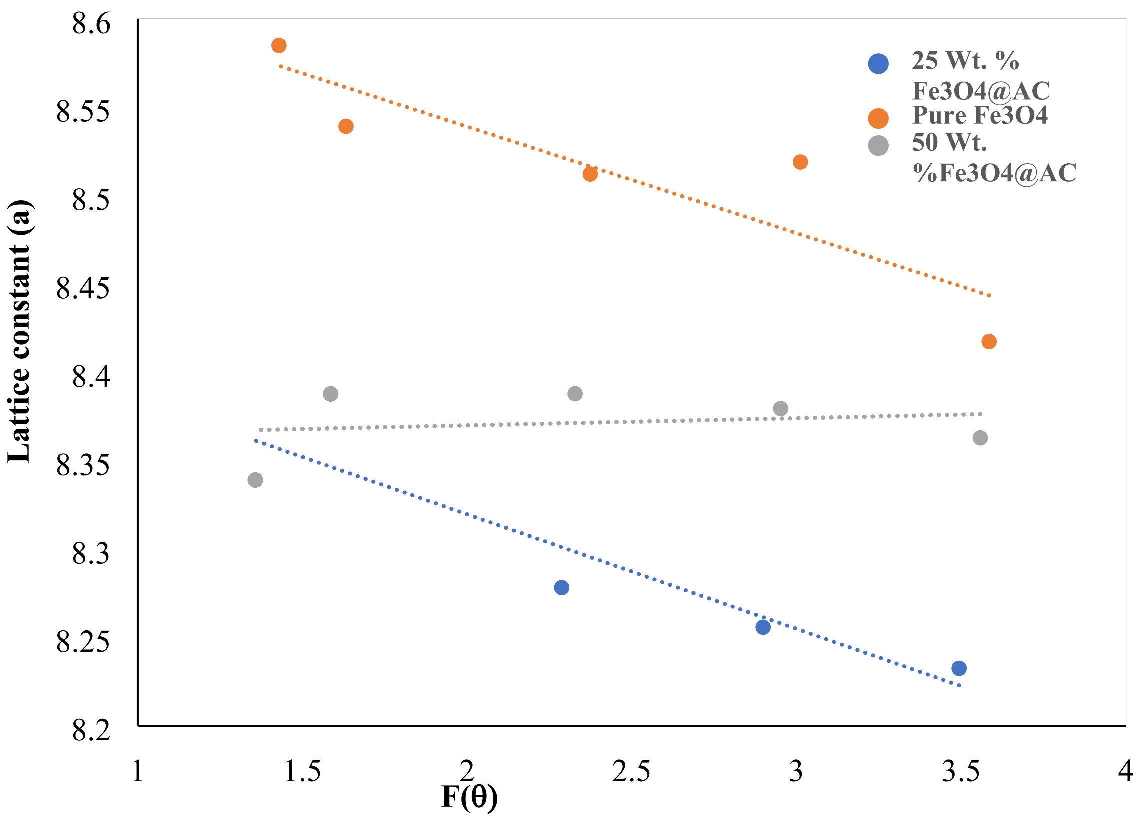

Fig. S4 Nelson-Riley (NR) plot of supported and non-supported catalysts

References

[1] S.V. Niveditha, R. Gandhimathi, *Chemosphere* 2020, **242,** 125189.

[2] A. Tolba, M.G. Alalm, M. Elsamadony, A. Mostafa, H. Afify, D. Dionysiou, *Process Saf. Environ.* 2019, **128,** 273-283.
